# A Comparative Study of Rapid Fresh Pathology Imaging and Standard FFPE H&E Histopathology: A High Concordance in the Evaluation of Lung and Breast Cancer

**DOI:** 10.3390/diagnostics16101503

**Published:** 2026-05-15

**Authors:** Min-Shu Hsieh, Yao-Chen Tseng, Chung-Yen Huang, Hsuan Wang, Yi-Hsuan Lee, Huang-Chun Lien, Hsin-Yi Huang, Koping Chang, Huan-Chih Wang, Kuo-Chuan Wang, Ming-Yang Wang, Yi-Hua Liao, Chi-Kuang Sun, Jin-Shing Chen

**Affiliations:** 1 Department of Pathology, National Taiwan University Cancer Center, Taipei 106319, Taiwan; a00302@ntucc.gov.tw; 2 Graduate Institute of Photonics and Optoelectronics and Department of Electrical Engineering, National Taiwan University, Taipei 106319, Taiwan; opiuy1234567@yahoo.com.tw (Y.-C.T.); sun@ntu.edu.tw (C.-K.S.); 3 Department of Pathology, National Taiwan University Hospital, Taipei 100225, Taiwan; bluesky12692005@gmail.com (C.-Y.H.); yihsuan65@gmail.com (Y.-H.L.); huangchunlien@ntu.edu.tw (H.-C.L.); neuropathology.ntuh@gmail.com (H.-Y.H.); c.koping@gmail.com (K.C.); 4 Department of Surgery, National Taiwan University Hospital, Taipei 100225, Taiwan; jessehcwang@ntu.edu.tw (H.-C.W.); wang081466@yahoo.com.tw (K.-C.W.); 5 Division of Breast Surgery, National Taiwan University Cancer Center, Taipei 106319, Taiwan; suryang1971@hotmail.com; 6 Department of Dermatology, National Taiwan University Hospital, Taipei 100225, Taiwan; yihualiao@ntu.edu.tw; 7 Graduate Institute of Biomedical Electronics and Bioinformtics and Institute of Medical Device and Imaging, National Taiwan University, Taipei 106319, Taiwan; 8 Research Center for Applied Sciences, Academia Sinica, Taipei 115201, Taiwan

**Keywords:** rapid fresh pathology, tumor assessment, digital histopathology, lung cancer, breast cancer

## Abstract

**Background/Objectives**: An intraoperative pathological assessment is essential for surgical decision-making but is limited by artifacts and a suboptimal image quality in frozen section analysis (FSA). Rapid fresh pathology (RFP), enabled by whole-mount tissue staining and a digital H&E transformation, has emerged as a potential alternative. This study aims to clinically validate the feasibility and diagnostic performance of RFP in lung and breast surgical specimens. **Methods**: Fresh surgical specimens, including tumor and adjacent non-neoplastic tissues, from patients with lung and breast lesions were processed using a rapid staining protocol and optical imaging via PATHOscope PS100 (Mesoview, New Taipei City, Taiwan) without slicing tissues. Diagnoses based on RFP images were independently evaluated by pathologists and compared with reference diagnoses obtained from formalin-fixed paraffin-embedded FFPE tissue histology to assess diagnostic concordance. **Results**: RFP demonstrated a high concordance with standard histopathology in both lung and breast specimens. The technique preserved the tissue architecture and provided a clear visualization of the cellular morphology, similar to conventional FFPE processing. In addition, RFP significantly reduced the time required for the tissue evaluation compared with FFPE workflows. **Conclusions**: RFP is a feasible and reliable approach for the rapid evaluation of lung and breast surgical specimens. Its ability to provide high-quality morphological information while preserving tissue integrity highlights its potential for future clinical applications, including intraoperative and time-sensitive pathological assessments.

## 1. Introduction

An accurate intraoperative pathological assessment is essential for guiding surgical decision-making across a wide range of malignancies, especially in the lung and breast [1,2,3,4,5,6]. The ability to rapidly determine tumor margins, identify metastatic involvement, and distinguish benign from malignant tissues directly influences operative strategies and patient outcomes [1,2,3,4,5,6]. Currently, frozen section analysis (FSA) remains the standard method for intraoperative diagnosis. However, despite its widespread adoption, FSA is inherently constrained by technical and practical limitations. The freezing process of FSA can cause variable tissue artifacts, such as nuclear smudging, blurred chromatin, distorted cell structures, and tissue folding or overlapping, especially in adipose-rich tissues [7,8,9]. Furthermore, FSA requires on-site well-trained technicians and experienced pathologists to provide a timely intraoperative diagnosis. Moreover, tissue loss is inevitable during FSA, which may limit subsequent histopathological or molecular analyses [9].

These limitations highlight an unmet need for rapid pathological approaches that can provide reliable, high-quality morphological information without compromising tissue integrity. In response, a variety of emerging techniques have been developed to enable rapid or slide-free histological assessments [10,11,12]. These approaches often leverage optical imaging and computational methods to visualize fresh tissue without conventional fixation and sectioning. While such techniques have demonstrated promising results, challenges remain in achieving consistent morphological fidelity, particularly when alternative staining strategies or non-standard contrast mechanisms are employed. Ensuring compatibility with conventional hematoxylin and eosin (H&E)-based interpretations remains an important consideration for clinical adoption.

Rapid fresh pathology (RFP) has recently emerged as a promising approach that integrates rapid whole-mount tissue staining, high-speed optical imaging, and a digital H&E transformation [13,14,15,16]. By avoiding freezing and sectioning, RFP minimizes tissue distortion and preserves the native architecture, while the optically sectioned high-resolution digital images from H&E-stained whole-mount tissues enable intuitive interpretation without requiring a substantial retraining of pathologists. These characteristics suggest that RFP may offer a practical and scalable solution to address the limitations of conventional intraoperative pathology. In this study, we systematically evaluate the applicability of RFP in surgically resected lung and breast surgical specimens [13,14,15,16]. We assess its diagnostic performance, morphological fidelity, and workflow efficiency in comparison with conventional intraoperative and standard histopathological approaches. Through this investigation, we aim to determine the potential of RFP as a pathology platform for real-time intraoperative pathology and its role in improving surgical decision-making for lung and breast cancers.

## 2. Materials and Methods

This study was a prospective clinical evaluation of rapid fresh pathology (RFP) for ex vivo tissue assessment. The study protocol was approved by the Research Ethics Committee of National Taiwan University (IRB number: 202405132RINE, approved on 20 June 2024), and all procedures were conducted in accordance with relevant guidelines and regulations.

### 2.1. Tissue Preparation

This study enrolled 20 patients undergoing surgical resection for lung adenocarcinoma (*n* = 10) or breast cancer (*n* = 10). Multiple tissue samples were collected from both tumor and non-tumor regions of the resected specimens. In total, 77 lung and 50 breast specimens were analyzed from the 10 cases of each cancer type (Table 1). Diagnostic performance was assessed at the specimen level, with each specimen representing an individual region of interest.

Fresh tumor and non-tumor specimens were first stained with hematoxylin (H9627, Sigma-Aldrich, Saint Louis, MO, USA) for 4 min. Following a thorough rinse, the tissues were immersed in an ammonia solution for bluing and were subsequently stained with eosin (3800, J.T.Baker, Phillipsburg, NJ, USA) for 12–15 s. The specimens were then rinsed with water for 10 s and 90% ethanol for 30 s prior to imaging. The entire staining procedure was completed within 7 min, facilitating rapid preparation of fresh tissues for subsequent optical imaging.

### 2.2. Optical Imaging via PATHOscope

**Figure 1:**
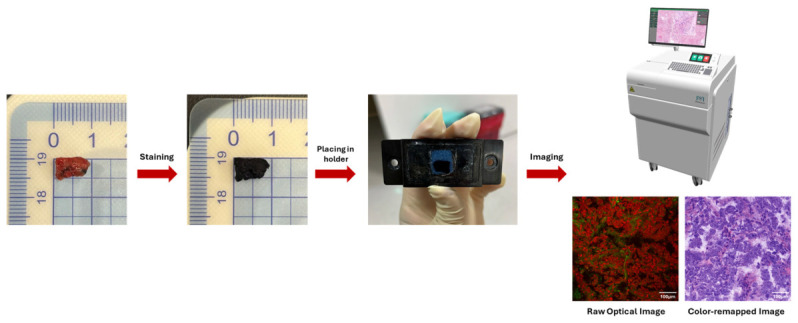
Workflow of rapid fresh pathology (RFP). Fresh tissue is rapidly stained, placed on a sample holder, and directly imaged to generate H&E-stained whole-mount tissue images from raw optical images after digital color transformation.

Following staining, specimens were imaged using the PATHOscope PS100 (MesoView, New Taipei City, Taiwan; FDA Establishment Registration No. 10093707), a high-speed optical imaging system optimized for large-area tissue acquisition (Figure 1). Stained fresh specimens were placed in a specialized holder under mild pressure to flatten the imaging surface, which was then positioned for scanning. Images were acquired across the entire specimen area and processed via image reconstruction, stitching, and digital color transformation to generate virtual hematoxylin and eosin (H&E) images. Dual-channel signals corresponding to nuclear and cytoplasmic contrast were integrated to achieve high-resolution morphological visualization (technical specifications available at: https://mesoview.com/pathoscope/ (accessed on 8 April 2026)). The H&E color transformation was performed using a previously reported method based on mapping intrinsic optical signals to H&E–histology contrast, without altering underlying structural information [13,17].

### 2.3. Validation of RFP Imaging Against Standard FFPE H&E-Stained Slides for Pathological Evaluation

Following RFP image acquisition, the fresh specimens were subjected to standard pathological processing to generate formalin-fixed paraffin-embedded (FFPE) tissue blocks. Two board-certified pathologists, blinded to the specimen identities and clinical data, independently reviewed both the FFPE H&E-stained slides and the corresponding RFP images for all lung and breast specimens. Case order was randomized during review to minimize potential order bias. Confidence intervals for diagnostic performance metrics were calculated using the exact Clopper–Pearson method. Diagnostic agreement was assessed using Cohen’s kappa coefficient. Kappa was calculated for interobserver agreement between the two pathologists based on RFP interpretations and for agreement between RFP diagnosis and the FFPE reference diagnosis. Diagnoses were categorized as benign/non-neoplastic or malignant.

Turnaround time for the RFP workflow was defined as the total time from tissue staining to final pathological interpretation. This included (1) staining time, (2) image acquisition time using the PATHOscope PS100 system (MesoView, New Taipei City, Taiwan), and (3) pathologist review time. It is noted that image acquisition time varied depending on specimen size. Pathological interpretation was performed under time-controlled conditions, with each case reviewed within a maximum of 5 min. This approach was intended to simulate real-world intraoperative workflows where rapid diagnostic decisions are required.

## 3. Results

### 3.1. RFP Results of Lung Specimens

**Figure 2:**
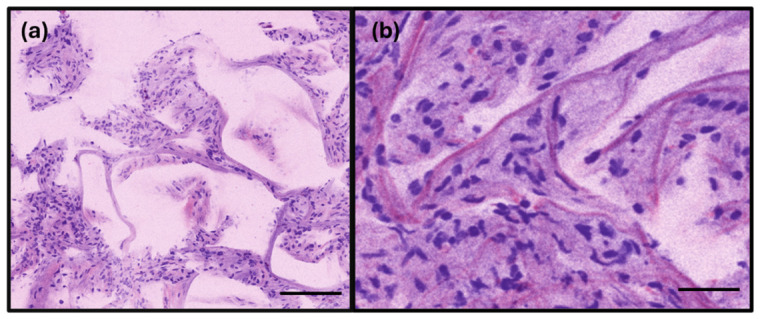
The rapid fresh pathology image of normal lung parenchyma. (**a**) Normal alveoli with thin capillaries and a flat pneumocyte lining. (**b**) The flat pneumocyte lining shows bland, nonoverlapping nuclei with a size similar to that of a small lymphocyte (scale bar: (**a**)—150 µm (**b**)—50 µm).

RFP images of non-neoplastic lung parenchyma revealed delicate alveolar walls lined by flat pneumocytes with small, bland nuclei (Figure 2). The alveolar wall capillaries were highly conspicuous, and bronchovascular bundles were readily identifiable. The bronchial epithelium appeared as a dense cellular layer, consistent with its characteristic pseudostratified morphology. Pulmonary arteries exhibited thick muscular walls lacking an epithelial lining, and intraluminal red blood cells were clearly visible. Alveolar macrophages appeared as discohesive cells characterized by an abundant eosinophilic cytoplasm, oval-to-round nuclei, and a low nuclear-to-cytoplasmic (N/C) ratio. Notably, anthracotic pigments were not discernible on RFP imaging.

**Figure 3:**
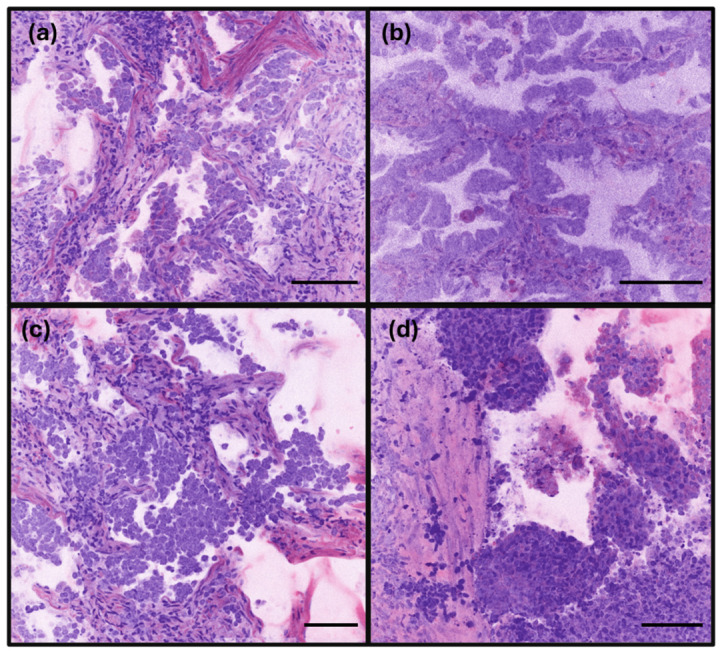
Rapid fresh pathology image of lung adenocarcinoma with different histologic patterns. (**a**) Lepidic pattern: Adenocarcinoma with large nuclei, increased cellularity with overlapping nuclei, and arranged along preserved alveolar structures. (**b**) Acinar pattern: Adenocarcinoma featuring large nuclei and nuclear overlapping arranged in glandular or acinar structures. (**c**) Micropapillary pattern: Adenocarcinoma presenting as multilayered cellular clusters consistent with a micropapillary growth pattern. (**d**) Solid pattern: Carcinoma cells with prominent, large nuclei arranged in cohesive solid nests (scale bar: 150 µm).

**Figure 4:**
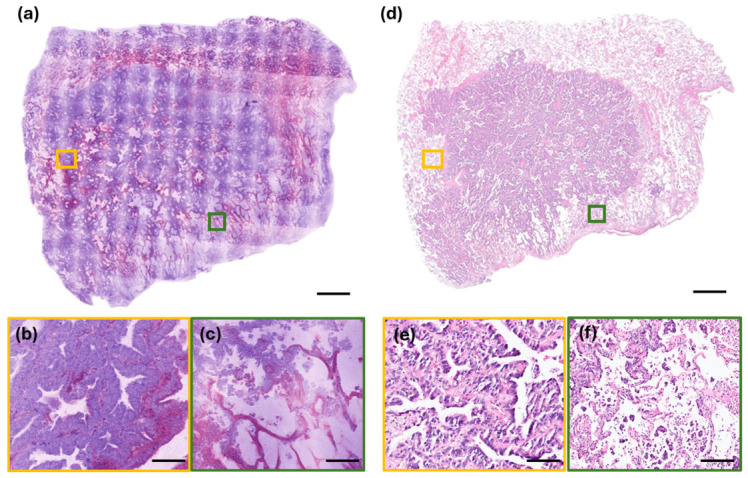
Comparative analysis of rapid fresh pathology (RFP) and FFPE histology in lung adenocarcinoma. (**a**–**c**) Rapid fresh pathology (RFP) images: (**a**) low-power view demonstrating detailed architectural features; (**b**) high-power view showing distinct cytological features of tumor cells; and (**c**) identification of tumor cells spreading through air spaces (STAS). (**d**–**f**) Corresponding FFPE H&E histology: (**d**) low-power architecture; (**e**) high-power cytological detail; and (**f**) histological confirmation of STAS (scale bars: (**a**,**d**) = 1 mm; (**b**,**c**,**e**,**f**) = 100 µm).

The low-power RFP images of lung adenocarcinoma demonstrated a marked increase in cellularity within the lining epithelium compared to non-tumor areas. Key cytological features—including enlarged nuclei (exceeding four to six times the size of small lymphocytes), coarse chromatin, conspicuous nucleoli, and nuclear overlapping—were clearly identifiable on high-power RFP images (Figure 3). While architectural patterns such as lepidic, acinar, micropapillary, and solid growth were well-preserved and easily observed, distinct cell borders were not clearly discernible. For lung adenocarcinoma, both the RFP and FFPE histology demonstrated comparable image details, including architectural and cytological features as well as tumor cells spreading through air spaces (STAS) (Figure 4).

### 3.2. RFP Results of Breast Specimens

**Figure 5:**
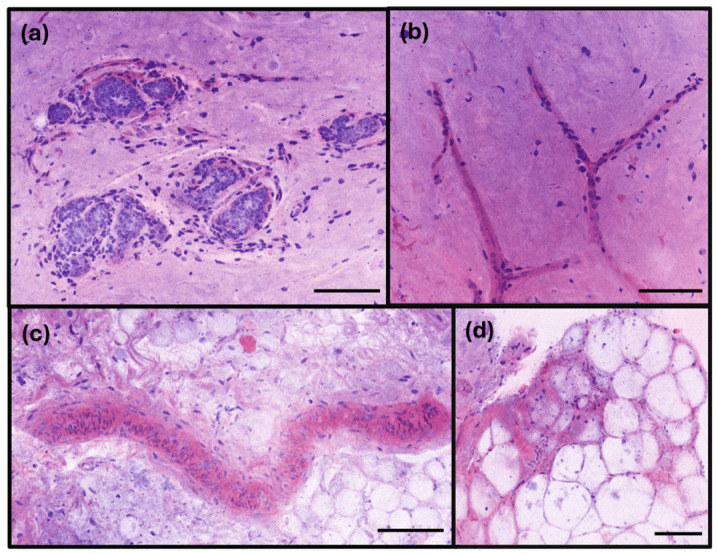
Representative benign breast tissue imaged using rapid fresh pathology (RFP). (**a**) Terminal duct lobular units (TDLUs) composed of small ducts and acini. The structures are lined by a distinct dual layer of epithelial and myoepithelial cells, embedded within a fibrous stroma containing scattered stromal cells. (**b**) Stromal microvasculature with delicate, branching capillary-like structures lined by flattened endothelial cells within the dense fibrous stroma. (**c**) Vessels with smooth muscle wall in a fibroadipose stroma. (**d**) Mature adipocytes characterized by clear cytoplasm and peripherally displaced nuclei, representing the normal fatty component of the mammary stroma (scale bar: 100 µm).

The RFP images of the non-tumor part of the breast revealed mammary glands with their typical ducts and terminal duct lobular units (TDLUs). The nuclei were small and bland. The collagen fibers, adipose cells, vessels, and capillaries in the background stroma were all clearly observed (Figure 5). Benign breast lesions like adenosis and fibrocystic disease were also observed on RFP images.

**Figure 6:**
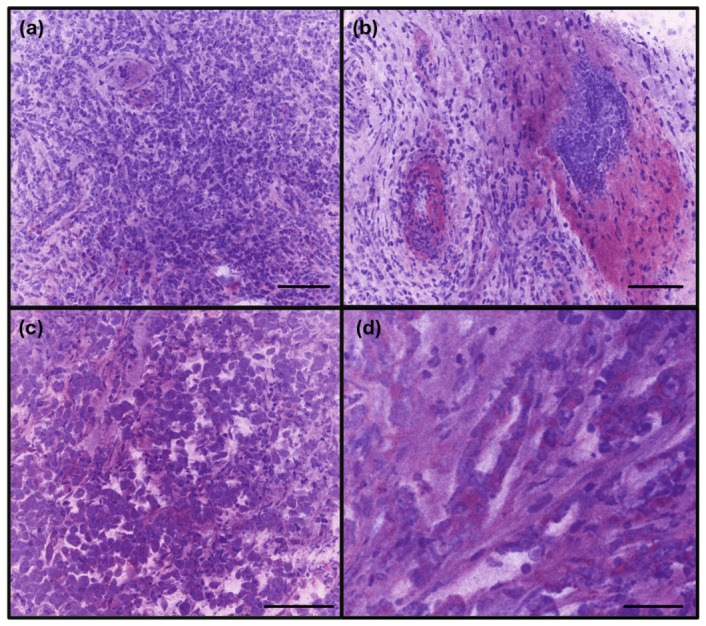
Representative malignant breast specimen imaged using rapid fresh pathology (RFP). (**a**,**b**) Tumor cells arranged in discohesive infiltrative patterns with irregular nuclei, imparting a lobular carcinoma-like growth pattern. (**c**) Solid nests and sheets of tumor cells with high cellular density and nuclear atypia, lacking clear glandular formation. (**d**) Areas showing glandular differentiation embedded within a desmoplastic stroma (scale bars: (**a**–**c**)—100 µm, (**d**)—50 µm).

**Figure 7:**
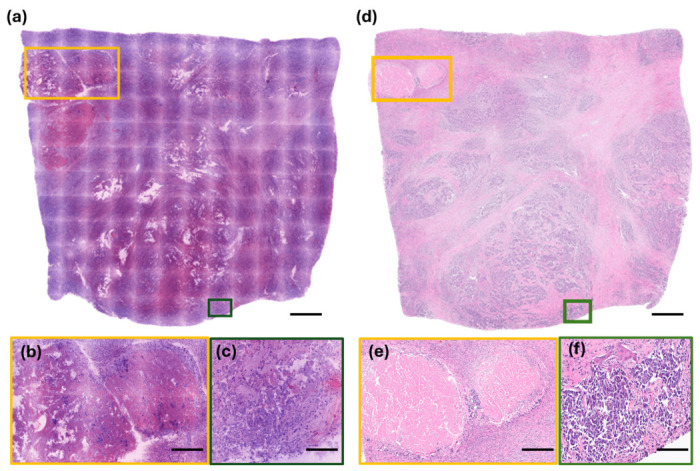
Comparative analysis of representative malignant breast specimens using rapid fresh pathology (RFP) and FFPE histology. (**a**,**d**) Low-power views of the same specimen acquired via (**a**) RFP and (**d**) corresponding FFPE H&E histology, demonstrating consistent tissue architecture across the entire specimen. Colored boxes indicate regions selected for high-magnification analysis. (**b**,**e**) Necrotic regions: high-magnification images of (**b**) RFP and (**e**) FFPE histology highlighting necrotic areas characterized by a loss of cellular detail and an amorphous, eosinophilic appearance. (**c**,**f**) Viable tumor regions: high-magnification views of (**c**) RFP and (**f**) FFPE histology showing viable neoplastic cells with well-preserved cellular and nuclear morphology (scale bars: (**a**,**d**) = 1 mm; (**b**,**e**) = 300 µm; (**c**,**f**) = 150 µm).

In contrast, the invasive breast carcinoma under RFP exhibited hypercellular lesions with a complete effacement of the normal breast parenchyma (Figure 6). The characteristic features of invasive lobular carcinoma—comprising discohesive cells with hyperchromatic, round nuclei arranged in infiltrating single-file patterns—were readily discernible (Figure 6a,b). Similarly, the invasive carcinoma of no special type (NST) on RFP images clearly demonstrated malignant cells with enlarged nuclei, increased N/C ratios, and conspicuous nucleoli, forming infiltrating nests or glandular structures within the adipose tissue and fibrotic stroma (Figure 6c,d); however, distinct cell borders were less apparent. For both subtypes of breast carcinomas, the RFP and FFPE histology provided a comparable morphological detail, including architectural patterns, cytological features, and the presence of tumor necrosis (Figure 7).

### 3.3. Rapid Fresh Pathology (RFP) as a High-Concordance Alternative to FFPE H&E Histopathology for Lung and Breast Cancer Diagnosis

The diagnostic performance of RFP was evaluated by comparing blinded interpretations of RFP images against reference diagnoses established from the FFPE histopathology by two pathologists. Across all specimens, RFP demonstrated a complete diagnostic concordance with the standard histopathology. Every case was classified as concordant between the RFP and FFPE diagnoses, with no minor or major discrepancies observed between the two pathologists.

For the detection of malignant lesions, RFP achieved a sensitivity, specificity, positive predictive value (PPV), and negative predictive value (NPV) of 100%. The overall diagnostic accuracy was 100%. These performance metrics were calculated based on true positive (TP), true negative (TN), false positive (FP), and false negative (FN) classifications, as summarized in the consolidated results of the lung and breast specimen cohorts (Table 2). Using the exact Clopper–Pearson method, the 95% confidence intervals were 96.6–100% for sensitivity, 92.8–100% for specificity, and 97.7–100% for overall accuracy. Cohen’s kappa analysis demonstrated an agreement between the two pathologists for RFP interpretation (κ = 1.00). The agreement between the RFP-based diagnosis and the FFPE reference diagnosis was also perfect (κ = 1.00).

Notably, RFP enabled the reliable differentiation of benign and malignant tissues across both lung and breast specimens, demonstrating a strong diagnostic performance despite inherent differences in the tissue composition and structural characteristics. Regardless of the specific organ origin, the system maintained 100% accuracy when identifying adenocarcinoma in lung specimens and carcinoma in breast specimens.

The image acquisition time for RFP ranged from approximately 1 to 4 min, depending on the specimen size. The pathologists’ interpretation was rapid, with an average evaluation time of approximately 1–2 min per case.

## 4. Discussion

This comparative study showed a high concordance between RFP imaging and standard FFPE H&E-stained slides. RFP can produce images from H&E-stained tissue without tissue sectioning. The RFP image effectively demonstrates the architecture and cytological features of both tumor and non-tumor parts of lung and breast specimens, supporting its feasibility as a rapid histopathological assessment approach.

RFP has advantages and challenges compared with traditional FFPE histopathology. RFP provides rapid, on-site diagnostic capability without requiring tissue freezing or sectioning and functions without the need for specialized facilities or highly trained histotechnicians. However, certain morphological features may not be as clearly demonstrated on RFP images as they are in permanent sections. First, cellular borders and cytoplasmic details are less defined, a limitation likely stemming from the absence of physical thin sectioning and the unique nature of optical contrast mechanisms. Second, anthracotic pigments—which are prominent in FFPE lung specimens—may appear inconspicuous on RFP, potentially due to differences in signal generation. Nevertheless, these differences did not affect the diagnostic interpretation in this study. RFP provides clear architectural features at a low power, and no diagnostic discrepancies were observed between the RFP and FFPE evaluation.

The use of mild pressure to flatten fresh tissue may introduce potential structural distortion, particularly in soft or delicate tissues. However, in this study, only minimal pressure was applied to achieve a stable imaging surface. Importantly, key architectural features relevant to diagnosis, including tumor growth patterns and tissue organization, were preserved and consistently identifiable. No diagnostic discrepancies were observed between the RFP and FFPE evaluation, suggesting that the applied compression did not adversely affect diagnostic reliability.

At our institution, lung and breast tumors constitute the majority of intraoperative frozen section consultations. Most submitted lung lesions are CT-detected ground-glass nodules that have not undergone a preoperative biopsy. In breast surgery, surgeons frequently request intraoperative assessments of surgical margins, nipple margins, and the sentinel lymph node status. The high concordance between RFP and FFPE H&E-stained slides observed in this study suggests that RFP is a promising modality for intraoperative diagnosis. This technology may be particularly valuable for hospitals lacking the specialized personnel or infrastructure required to maintain a standard frozen section service.

Several approaches have been developed to enable the rapid or slide-free histological assessment of fresh tissues [10,11,12]. Some methods rely on alternative staining strategies or intrinsic optical contrast, which may produce image appearances that differ from conventional hematoxylin and eosin (H&E) staining and require adaptation for interpretation. Other imaging-based techniques have demonstrated a promising morphological visualization; however, these studies often emphasize instrument-level performance, such as the scanning speed or exposure time, without clearly reporting the total workflow from specimen preparation to diagnostic interpretation.

The present study has several limitations that should be acknowledged. First, the current validation was restricted to lung and breast specimens; therefore, further studies are required to evaluate the broader applicability of RFP across additional organ systems. Second, although this study demonstrated a high concordance for identifying malignancies, benign inflammatory and infectious conditions were not systematically included and warrant dedicated investigation in the future. Finally, variations in mechanical tissue properties in different organs influenced the image acquisition. This necessitated specific adjustments to sample preparation protocols to ensure a consistent image quality across the different tissue types encountered. This study showed that RFP can provide rapid H&E contrast images and demonstrated its potential as a complementary approach to frozen section analysis for rapid intraoperative assessments. Further studies comparing RFP and frozen sections are needed and ongoing.

A summary comparison of RFP and FFPE histopathology is provided in Table 3. RFP offers a rapid, non-destructive imaging approach that preserves the tissue architecture without the need for freezing or physical sectioning. While cytological details may be less distinct compared to FFPE, RFP enables the fast and reliable identification of key morphological features. FFPE remains the gold standard for histopathological diagnosis, offering the highest structural and cytological fidelity at the expense of a longer processing time.

## 5. Conclusions

This validation study demonstrates a 100% diagnostic concordance between RFP imaging and standard FFPE H&E-stained slides for both lung and breast specimens. The critical architectural and cytological features of these malignancies were readily identifiable across both low- and high-power RFP magnifications. Consequently, RFP imaging represents an efficient, rapid, and labor-saving modality with significant potential to enhance intraoperative pathological consultations, particularly in settings with limited personnel.

## 6. Patents

The technology described in this study is related to a patent application entitled “A rapid staining method for pathological specimens”, filed by National Taiwan University, which is currently under review.

## Figures and Tables

**Table 1:** Sampled lung and breast specimens submitted for rapid fresh pathology.

Specimen Type	Lung	Breast	Total
Patients (*n*)	10	10	20
Tumor part (*n*)	53	34	87
Non-tumor part (*n*)	24	16	40
Total specimens (*n*)	77	50	127

**Table 2:** Diagnostic concordance between rapid fresh pathology (RFP) and formalin-fixed paraffin-embedded (FFPE) examination in lung and breast specimens.

Specimen Type	Pathologist 1		Pathologist 2	
Lung	FFPE Benign	FFPE Malignant	FFPE Benign	FFPE Malignant
RFP Benign	24	0	24	0
RFP Malignant	0	53	0	53
Breast				
RFP Benign	16	0	16	0
RFP Malignant	0	34	0	34
Total	40	87	40	87

**Table 3:** Comparison of rapid fresh pathology (RFP) and formalin-fixed paraffin-embedded (FFPE) pathology in terms of workflow, imaging characteristics, tissue preservation, and clinical application.

Parameter	Rapid Fresh Pathology (RFP)	Formalin-Fixed Paraffin-Embedded Pathology (FFPE)
Sample preparation	No freezing or physical sectioning required	Requires fixation, embedding, sectioning
Image acquisition	Optical imaging (no physical sectioning)	Physical thin section
Tissue integrity	Preserved (no cutting artifacts)	High structural fidelity
Architectural preservation	Excellent	Excellent
Clinical application	Rapid assessment	Gold-standard diagnosis
Limitations	Reduced cytological clarity in some cases	Long processing time

## Data Availability

The data supporting the findings of this study are available from the corresponding authors upon reasonable request. The data are not publicly available due to privacy and ethical restrictions.
